# Effects of combined dredging-related stressors on sponges: a laboratory approach using realistic scenarios

**DOI:** 10.1038/s41598-017-05251-x

**Published:** 2017-07-12

**Authors:** Mari-Carmen Pineda, Brian Strehlow, Jasmine Kamp, Alan Duckworth, Ross Jones, Nicole S. Webster

**Affiliations:** 1 Australian Institute of Marine Science (AIMS), Townsville, QLD and Perth, WA Australia; 2 Western Australian Marine Science Institution, Perth, WA Australia; 3 School of Biological Sciences, Centre for Microscopy Characterisation and Analysis, and Oceans Institute, University of Western Australia, Crawley, WA Australia; 4 James Cook University, Townsville, QLD Australia

## Abstract

Dredging can cause increased suspended sediment concentrations (SSCs), light attenuation and sedimentation in marine communities. In order to determine the combined effects of dredging-related pressures on adult sponges, three species spanning different nutritional modes and morphologies were exposed to 5 treatment levels representing realistic dredging scenarios. Most sponges survived under low to moderate turbidity scenarios (SSCs of ≤ 33 mg L^−1^, and a daily light integral of ≥0.5 mol photons m^−2^ d^−1^) for up to 28 d. However, under the highest turbidity scenario (76 mg L^−1^, 0.1 mol photons m^−2^ d^−1^) there was 20% and 90% mortality of the phototrophic sponges *Cliona orientalis* and *Carteriospongia foliascens* respectively, and tissue regression in the heterotrophic *Ianthella basta*. All three sponge species exhibited mechanisms to effectively tolerate dredging-related pressures in the short term (e.g. oscula closure, mucus production and tissue regression), although reduced lipids and deterioration of sponge health suggest that longer term exposure to similar conditions is likely to result in higher mortality. These results suggest that the combination of high SSCs and low light availability can accelerate mortality, increasing the probability of biological effects, although there is considerable interspecies variability in how adult sponges respond to dredging pressures.

## Introduction

Sediments released into the water column by natural resuspension, river runoff and human activities such as dredging pose a potential risk to sensitive ecosystems such as coral reefs, seagrass meadows and sponge gardens^1–4^. Sediments in suspension, or settling back out of suspension (i.e. sedimentation), can affect epi-benthic organisms in a number of ways, including clogging of the feeding and filtering mechanisms. Water turbidity (cloudiness) can also temporarily reduce or extinguish benthic light^4–6^. These stressors can act alone but more often in combination, making impact prediction particularly difficult. Sponges are sessile filter-feeding organisms that play important roles in marine ecosystems, including substrate consolidation, habitat provision, seawater filtration and bentho-pelagic energy transfer^7–9^. Despite their abundance and ecological importance, our understanding of how sponges respond to turbidity is still very basic^10, 11^. This knowledge gap poses significant challenges to their effective management, especially for anthropogenic turbidity-generating activities such as dredging, that can at least be subject to some regulation and control^12^.

Most sponges obtain energy heterotrophically, by filtering seawater through an aquiferous system or internal canal network^13, 14^. However, many sponges can also obtain energy autotrophically from photosymbionts, of which *Cyanobacteria* are the most common^15, 16^. Some bioeroding sponge species also harbour dinoflagellates of the genus *Symbiodinium* as photosymbionts^17, 18^. The photosymbionts can provide >50% of the energy requirement of the host for some tropical sponge species^15, 19^. Overall, the diverse community of symbiotic microorganisms can comprise up to 35% of the sponge biomass and make other valuable contributions to many aspects of the sponge’s physiology and ecology (e.g. production of secondary metabolites)^20^. These microbial associations tend to be highly host specific, and are generally stable across broad geographic and environmental gradients^21^. This stable host-microbe consortium is often referred to as the ‘sponge holobiont’^20^.

Sponges can be affected by dredging-related pressures in different ways depending on their nutritional mode, their morphology and their behavioural and physical adaptations to tolerate sediment. For instance, due to their high filter-feeding activity, heterotrophic sponges may be affected by elevated suspended sediment concentrations (SSCs), with long term exposure to high SSCs clogging their aquiferous systems and reducing the flow of oxygenated seawater to the mesohyl^10^. In addition, phototrophic sponges may be affected by the reduction in benthic light availability that occurs in sediment plumes^5, 6^. Sediment deposition could also cause smothering and suffocation of recruits and adult sponges, especially encrusting, massive, cups and plate-like morphologies^10, 11, 22, 23^. All these effects can have flow-on consequences for host energetics, health and reproductive output^10, 11^.

Nevertheless, some sponges can tolerate, and in some case thrive in, turbid environments, including endopsammic species which live partially buried within sediments^10, 11, 24^. Knowing how they tolerate these conditions is important for understanding the consequences of turbidity generating activities. Some sponges can temporarily tolerate high SSCs through changes in their physiology, such as temporarily closing or reducing the size of their incurrent openings (ostia) or arresting pumping activity^25–28^. Phototrophic species may be able to temporarily tolerate low light by photoacclimation and an increase in photosynthetic efficiency^10, 29, 30^. To tolerate elevated sedimentation, some species have active cleaning mechanisms to remove sediments such as the production of mucus-like substances and tissue sloughing, selective rejection of inhaled particles and the use of water jets to unblock inhalant pores^10, 11, 24^. These ‘active’ mechanisms (requiring energy expenditure) work in conjunction with more ‘passive’ mechanisms that reduce sediment accumulation such as the existence of self-cleaning surfaces, and micro and macro morphology and orientation that promotes sediment rejection under gravitational forces^11, 31^. The removal of sediment by epibionts is also a passive mechanism for self-cleaning that has been reported in some sponges^11^.

Understanding physiological tolerance levels of sponges to turbidity and establishing dose-response relationships is essential for impact prediction purposes, i.e. to forecast the potential effect of turbidity-generating activities such as dredging at the environmental impact assessment stage. It is also essential for managing water quality during dredging, i.e. to alert dredging proponents to conditions which could harm local sponge populations. A key problem is that there are many different cause-effect pathways whereby sediment released into the water column can affect sponges and these cause-effect pathways may act alone or in combination, potentially confounding attempts to establish a dose and subsequent response in laboratory based or field studies^4, 32^. Elevated turbidity is clearly a hazard to sponge communities, as has been shown in many previous laboratory and field based studies^10, 11^, but whether it constitutes a risk depends on being able to place the results in the context of likely exposure scenarios. Recently, the spatial and temporal changes in water quality have been comprehensively described for several large scale dredging projects^5, 6, 33^. These capital projects occurred in a range of marine settings from inshore turbid reef to offshore ‘clear water’ environments, providing a suitable spectrum of conditions to describe the range of likely exposure scenarios^5, 6^. The analyses included an examination of pre-dredging (baseline) data, and data collected at various distance from the dredging (excavation) activities, allowing characterization of spatial patterns and effects relative to background conditions (i.e. natural resuspension events).

Water quality information from these dredging programs has already been used in a sequence of laboratory-experiments to better understand the risks posed to sponges by individual stressors. Sponges were exposed to a range of different light intensity treatments (range: 0–8.1 mol photons m^−2^ d^−1^) under very low SSCs (<0.05 mg L^−1^), so that light reduction was the primary variable examined^34^. Subsequently, sponges were exposed to a range of different SSC treatments (range: 0–76 mg L^−1^) and light was standardized across the treatments (to 5 mol photons m^−2^ d^−1^), such that SSC was the only variable being examined^35^. Finally, to derive pressure response values to sedimentation, sponges were repeatedly exposed to multiple discrete sediment deposition events (of 30−45 mg cm^−2^)^36^. These experiments were conducted with 5−6 sponge species, spanning heterotrophic and phototrophic nutritional modes, and a range of different morphologies. Endpoints included a range of physiological metrics encompassing respiration, survivorship, growth, lipid and chlorophyll content, production of mucus-like substances and changes in the microbial community (microbiome) composition. All experiments were conducted for an extended (chronic) time frame of 28 d, and included a 14 d post exposure monitoring or ‘observational’ period to see if there were any latent effects.

These studies showed that reducing the light levels clearly affected the phototrophic sponges^34^, with little effect on the heterotrophic species. Among phototrophs, the boring sponge *Cliona orientalis* and the cup-shaped *Carteriospongia foliascens* rapidly bleached (discoloured) when held in the dark. *C*. *orientalis* survived the 28 d exposure period, rapidly regaining colour when returned to normal light levels; in contrast, *C*. *foliascens* died in the observational period. Less extreme light reduction treatments (0.8 mol photons m^−2^ d^−1^) resulted in some minor bleaching but no mortality in either species. Microbial community composition did not differ between light treatments for the heterotrophic species, but did for the two phototrophic species that bleached. In the sediment exposure experiments, high SSCs alone resulted in negative growth, decreased respiration rates and lipid depletion in most sponges^35^. Significant necrosis and mortality occurred within most species, including *C*. *foliascens* and *C*. *orientalis* at 70 mg L^−1^, while no mortality occurred at lower SSCs (<23 mg L^−1^).

These studies are useful for identifying the physiological and behavioural responses specific to individual stressors, and for understanding the different susceptibility of species and nutritional modes. They also help in the interpretation of experiments when the stressors are combined — which is the subject of the current study. In this investigation, three different sponge species were exposed to five different treatments (referred to as ‘scenarios’) of elevated turbidity (i.e. combinations of elevated SSCs and associated reductions in light). As with the earlier experiments examining the individual stressors in isolation^34–36^, the experiments were conducted over a 28 d exposure period and a subsequent 14 d observational period. The SSCs and light levels used are based on the empirical data collected during several large scale capital dredging projects^5, 6^, allowing the results to be contextualized in a management framework.

## Results

### Physical parameters

**Figure 1:**
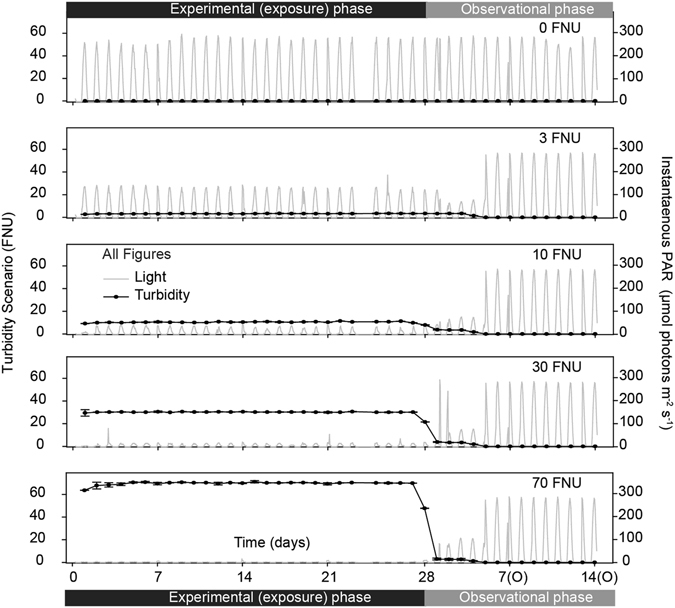
Physical parameters throughout the experiment. Turbidity (FNU) (mean ± SE) and instantaneous light (PAR, µmol photon m^−2^ s^−1^) (mean) (n = 2 replicate tanks per treatment) experienced in each scenario (0, 3, 10, 30 and 70 FNU, but see also Table 1) throughout the 28 d experimental (exposure) and 14 d observational periods.

Turbidity values as measured by the nephelometers (formazin nephelometric units, FNU) were constant (+1 FNU) throughout the experiment (Fig. 1), and there were significant differences between all treatments during the exposure period (Table 1, Table 2). Gravimetrically determined SSCs were also significantly different between treatments throughout the experiment (Repeated measures (RM) ANOVA: *P* < 0.001). Daily Light Integrals (DLIs) were also significantly different between treatments (Fig. 1, Table 1, Table 2). Mean sediment deposition (±SE), measured using SedPods, increased as FNU increased from 0.2−5.2 mg cm^−2^ d^−1^, respectively, and was significantly different between treatments during the experimental period (Table 1, Table 2). During the observational phase, all tanks returned to control conditions and no significant differences were retrieved between treatments (Fig. 1, Table 1, Table 2). The term ‘scenario’ in combination with the nominal turbidity value is used hereafter to refer to the treatments, and thus, for instance, the 70 FNU scenario refers to the SSC combination and DLI specified in Table 1 (76 mg L^−1^ and 0.15 mol photons m^−2^ d^−1^, respectively).

**Table 1:** Summary of exposure scenarios.

Turbidity Scenario	nephelometrically-derived SSC	Measured SSCs (gravimetrically determined)	Light (mid-day max)	Daily Light Integral (DLI)	SedPod accumulation rate
FNU	mg L^−1^	mg L^−1^	μmol photons m^−2^ s^−1^	mol photons m^−2^ d^−1^	mg cm^−2^ d^−1^
70	76	76.9 ± 2.6	12.3	0.15	5.2 ± 0.9
30	33	32.2 ± 2.2	18.7	0.46	3.3 ± 0.4
10	11	15.5 ± 1.8	35.4	0.87	2.5 ± 0.4
3	3	3.6 ± 0.4	119	3.07	1.1 ± 0.3
0	0	1.3 ± 0.3	267	6.34	0.2 ± 0.1
Observational	0	1.3 ± 0.3	271	6.53	0.2 ± 0.1

Turbidity (FNU), mean nephelometrically-derived SSC (mg L^−1^), measured SSCs determined gravimetrically at 7, 14, 21 and 28 d (mean ± SE, n = 3 samples per tank and day), maximum daily (mid-day) light level (instantaneous), daily light integral (DLI) and SedPod accumulation rate determined gravimetrically at 7, 14, 21 and 28 d (mean ± SE, n = 2 samples per tank and day), during the 28 d experimental period and subsequent 14 d observational period. Scenarios were based on *in situ* water quality data collected from three recent large scale capital dredging programs in Australia^5, 6^ (see Discussion).

**Table 2:** Analyses of variance on the effect of turbidity scenario on the physical parameters.

	Source	*df*	Turbidity (FNU)		Light (DLI)		SedPod accumulation rate (mg cm^−2^ d^−1^)	
			*F*	*P*	*F*	*P*	*F*	*P*
Experimental phase	Scenario	4	686.1	<0.001	212.1	<0.001	19.772	<0.001
	Error	65						
	Tukey		0 < 3 < 10 < 30 < 70		0 < 3 < 10 < 30 < 70		0, 3 < 10, 30 < 70	
Observational phase	Scenario	4	1.000	1.000	0.081	0.988	1.000	1.000
	Error	65						

One-way ANOVA on turbidity (FNU), light (DLI) and sedimentation data, at the end of the experimental and observational phase. Tukey tests have been performed for significant pairwise multiple comparisons between scenarios (scenarios: 0, 3, 10, 30 and 70 FNU).

### Sponge health, growth and stress responses to sediments

**Figure 2:**
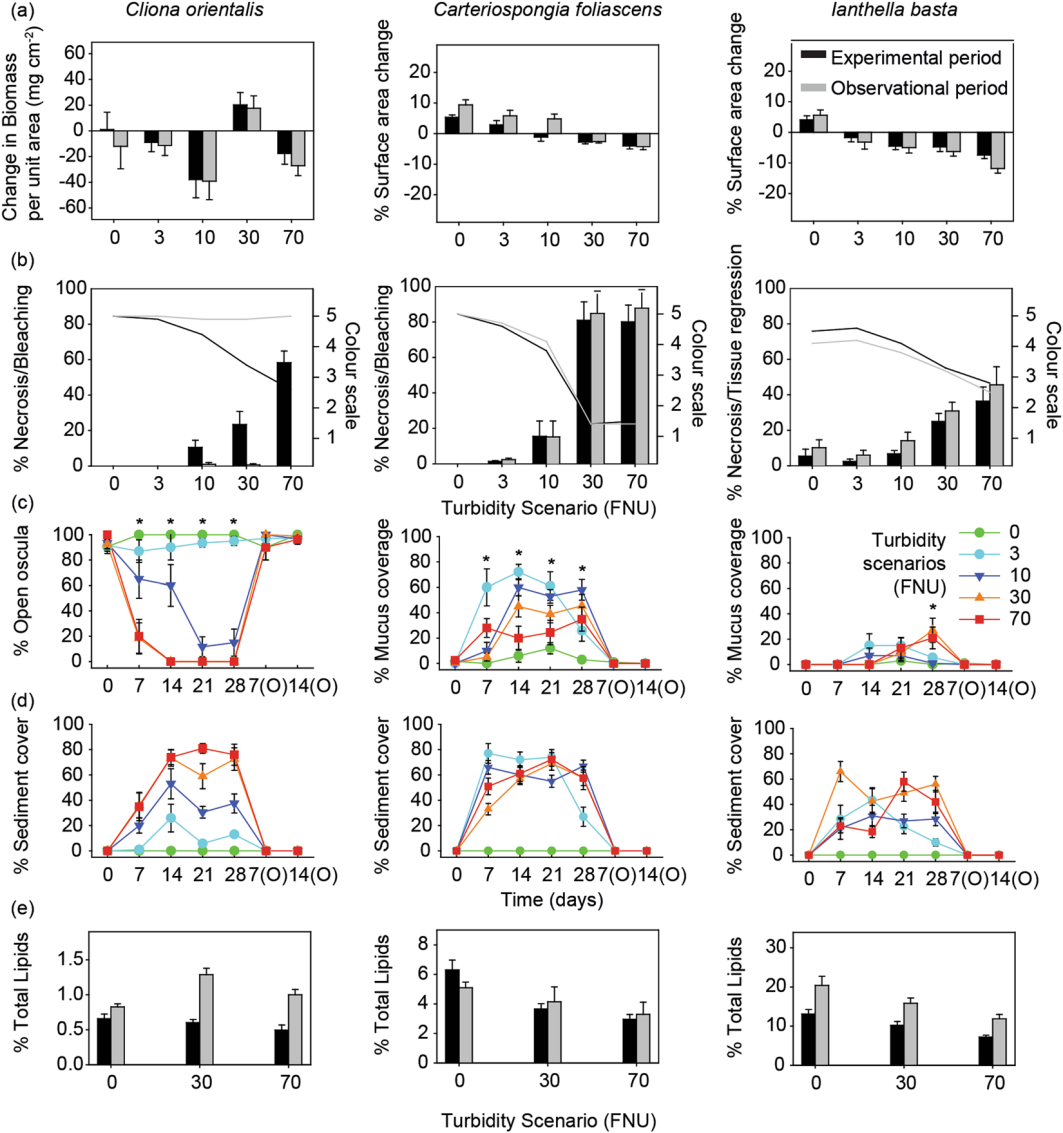
Sponge health, growth and behavioural responses. (**a**) Growth based on changes in biomass per unit area in *C*. *orientalis* (mg cm^−2^) and percentage change in surface area in *C*. *foliascens* and *I*. *basta*, (**b**) percentage of necrotic, bleached, or regressed tissue (bars) and colour scale (lines), (**c)** percentage of open oscula in *C*. *orientalis* and mucus production in *C*. *foliascens* and *I*. *basta* as stress responses to sediments, (**d**) percentage of sediment cover on sponges, and (**e**) percentage of sponge biomass comprised of lipids, for all species and targeted scenarios (0, 3, 10, 30 and 70 FNU) after the 28 d experimental period and 14 d observational period in **a**, **b** and **e** and throughout the experiment in c and d (mean ± SE). Asterisks in **c** show statistically significant differences between scenarios (ANOVA: *P* < 0.05).

**Figure 3:**
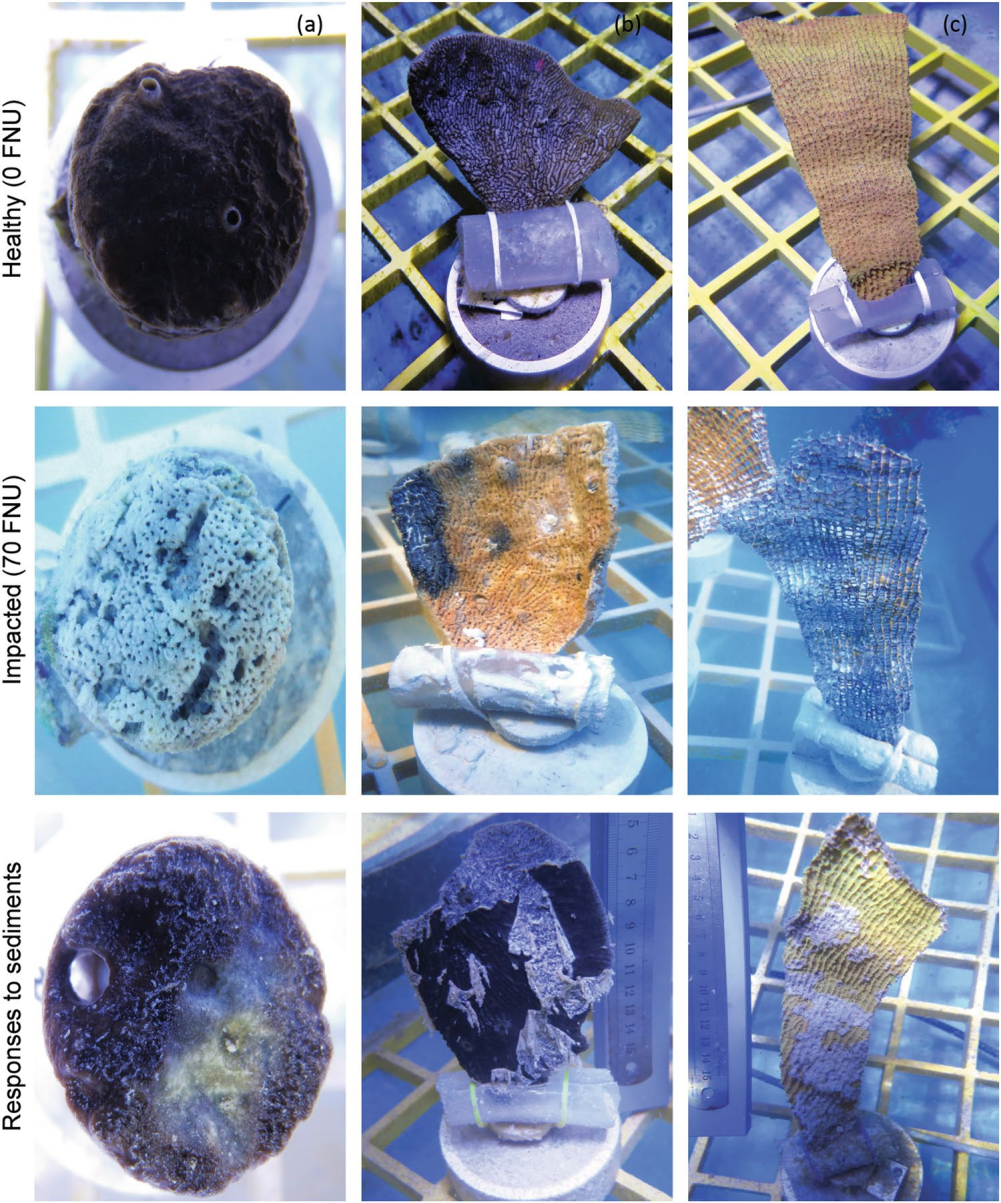
Sponge behavioural responses to sediments. (**a**) *C*. *orientalis*, (**b**) *C*. *foliascens*, and (**c**) *I*. *basta*. Healthy and impacted sponges under the control (0 FNU) and highest turbidity scenario (70 FNU), respectively, and their visual responses to sediment stress (i.e. oscula closure, mucus production and tissue regression).

Overall sponge health was negatively affected by the scenarios ≥ 10 FNU although the extent of the impact and the ability to recover during the observational phase were species-specific. Changes in biomass per unit area (mg cm^−2^) in *Cliona orientalis*, which are related to its bioerosion rate, varied greatly among scenarios. During the experimental period, the decrease in biomass in this species was higher under 3, 10 and 70 FNU scenarios, suggesting a continuous bioeroding activity (Fig. 2). The increase in biomass in the 30 FNU scenario was unexpected, and possibly due to an accumulation of sediments within the cores of some replicates, particularly in the necrotic regions which exposed the rugose and porous coral substrate and could accumulate sediments (see Fig. 3a) Overall, the variability among replicates was very high, and significant differences were only detected between the 10 and 30 FNU scenarios (Table 3a). For *Carteriospongia foliascens* and *Ianthella basta*, growth based on surface area throughout the experiment (i.e. experimental plus observational periods) had a negative relationship with increasing FNU, with greatest regression from both species at the 70 NTU scenario (Fig. 2a, Table 3a). Positive growth was only detected in *C*. *foliascens* in the ≤ 3 FNU scenarios, while negative growth occurred in the ≥ 30 FNU scenarios, during both the experimental and observational periods. *I*. *basta* showed negative growth under all dredging scenarios except for the control (Fig. 2a, Table 3a).

**Table 3:** Analyses of variance on the effects of dredging on the physiological responses of sponges.

Parameter	Phase	Source	*df*	*Cliona orientalis*		*Carteriospongia foliascens*		*Ianthella basta*	
				*F*	*P*	*F*	*P*	*F*	*P*
(**a**) growth	Expt.	Scenario	4	3.864	0.009	14.348	<0.001	10.674	<0.001
		Error	45						
		Tukey		10 < 30		0, 3 > 10, 30, 70		0 > 3, 10, 30, 70	
	Obs.	Scenario	4	3.116	0.024	17.623	<0.001	12.283	<0.001
		Error	45						
		Tukey		10 < 30		0, 3, 10 > 30, 70		0 > 3, 10, 30, 70	
(**b**) % necrosis, bleaching & tissue regression	Expt.	Scenario	4	39.639	<0.001	31.694	<0.001	31.435	<0.001
		Error	45						
		Tukey		0, 3 < 30, 70		0, 3, 10 < 30, 70		0, 3 < 30, 70	
	Obs.	Scenario	4	3.063	0.547	36.878	<0.001	22.751	<0.001
		Error	45						
		Tukey				0, 3, 10 < 30, 70		0, 3 < 30, 70	
(**c**) stress responses	Expt.	Scenario	4	103.028	<0.001	7.439	<0.001	19.567	<0.001
		Error	45						
		SNK		0, 3 > 10, 30, 70		0, 3 < 10, 30		0, 3, 10 < 30, 70	
	Obs.	Scenario	4	0.367	0.831	1.000	1.000	1.000	1.000
		Error	45						
(**d**) % lipids	Expt.	Scenario	2	1.709	0.200	13.913	<0.001	10.400	<0.001
		Error	27						
		SNK				0 > 30, 70		0 > 30 > 70	
	Obs.	Scenario	2	9.874	<0.001	1.292	0.291	6.293	0.006
		Error	27						
		SNK		0 < 30 > 70				0 > 70	

(a) One-way ANOVA on sponge growth (changes in biomass per unit area in *C*. *orientalis*, and relative growth rate based on surface area in *C*. *foliascens* and *I*. *basta*), (b) One-way ANOVA on ranks of the percentage of necrotic, bleached and regressed tissue, (c) One-way ANOVA on percentage of open oscula in *C*. *orientalis* and on percentage of mucus coverage in *C*. *foliascens* and *I*. *basta*, (d) One-way ANOVA on the percentage of sponge biomass comprised of lipids, for each species separately, at the end of the experimental and observational phase. Tukey and Student Newman-Keuls (SNK) tests have been performed for significant pairwise multiple comparisons in a-b and c-d, respectively (scenarios: 0, 3, 10, 30 and 70 FNU).

For *C*. *orientalis*, 20% of individuals (n = 4 replicates) died after 14−28 d in the 70 FNU scenario (Supplementary Fig. S1). While all remaining individuals in the ≥ 10 FNU scenarios had minor to moderate bleaching (discolouration) during the experimental period. These partially bleached sponges recovered their initial pigmentation during the observational phase (Fig. 2b). In contrast, *C*. *foliascens* was particularly sensitive, showing significantly higher rates of necrosis and mortality under the ≥ 30 FNU scenarios. Mortality affected 10, 55 and 85% of *C*. *foliascens* individuals in the 10, 30 and 70 FNU scenarios, respectively, with necrosis and mortality observed after 28, 21 and 7 d, respectively (Fig. 2b, Supplementary Fig. S1). No mortality was evident in the heterotrophic *I*. *basta* throughout the experiment; however some individuals had partial mortality with most tissue lost in sponges exposed to ≥ 30 FNU (Fig. 2b, Table 3b).

Mortality data for *C*. *foliascens* was fitted to nonlinear regression curves to calculate the lethal concentration (LC) at which 50% (LC_50_) and 10% (LC_10_) of the population died. Nonlinear regression of the dose−response curve (R^2^ = 0.9567, AICc = 48.11) met assumptions of normality and homoscedasticity and the replicates test showed no evidence for lack of fit (*P* = 0.694). After the 28 d exposure period, the LC_50_ (and 95% confidence intervals range) for mortality in *C*. *foliascens* was 47 mg L^−1^ (range: 40−56 mg L^−1^) and the LC_10_ was 22 mg L^−1^ (range: 14−31 mg L^−1^). These SSCs corresponded to an LC_50_ value for DLI of 0.3 mol photons m^−2^ d^−1^ (range: 0.21–0.33), and an LC_10_ DLI of 0.9 mol photons m^−2^ d^−1^ (range: 0.61–1.26 mol photons m^−2^ d^−1^). Mortality data from *C*. *orientalis* did not meet model assumptions and LC values could not be calculated.

In addition to partial or total mortality, sponges showed different sublethal responses to dredging-related pressures. Visual assessments of *C*. *orientalis* throughout the experiment revealed a significantly higher percentage of open oscula in the lower turbidity scenarios (<10 FNU) from day 7 until the end of the experimental phase (ANOVA: *P* < 0.05, Fig. 2c), indicating reduced pumping activity in this species within the higher turbidity scenarios (≥10 FNU). However, all oscula appeared open again once sponges were returned to control conditions during the observational phase (Fig. 2c, Fig. 3a, Table 3c). In contrast, *C*. *foliascens* produced layers of mucus when exposed to any sediment (Fig. 2c, Fig. 3b). The percentage of mucus cover was significantly higher in the 10 and 30 FNU scenarios at the end of the experimental phase, although no mucus was produced during the observational period (Fig. 2c, Table 3c). *I*. *basta* produced a moderate amount of mucus when exposed to sediments (Fig. 3c); with a significantly higher percentage of mucus cover in samples from the 30 and 70 FNU scenarios at the end of the 28 d exposure period and no mucus production observed during the recovery period (Fig. 2c, Table 3c). In both species the mucus layers were observed to eventually slough off the sponge surface leaving clean tissue relatively free of sediments (Fig. 3b-c). The observed sublethal responses to sediments were directly related to the percentage of sediment cover observed on top of sponges during the experiment (Fig. 2d).

### Lipid analysis

No depletion of total lipid content per sponge biomass was detected in the control samples throughout the experiment (i.e. from Time 0 until the end of the observational phase, T-tests: P > 0.05 for all species). However, total lipid content was lower in all species exposed to the ≥ 30 FNU scenarios by the end of the exposure period, although this was not statistically significant in *C*. *orientalis* (Fig. 2e, Table 3d). The percentage of total lipids in *C*. *foliascens* decreased from 6.3% ± 0.66 (mean ± SE) in the control to 2.9% ± 0.32 in the 70 FNU scenario, and no significant recovery of lipid content occurred during the observational phase. Similarly, the percentage of total lipids in *I*. *basta* decreased from 13.1% ± 1.13 (mean ± SE) in the control to 7.2% ± 0.47 in the 70 FNU scenario. Some recovery of lipid content was evident in *I*. *basta* towards the end of the observational phase (Fig. 2e, Table 3d). Finally, *C*. *orientalis* exposed to the 30 and 70 FNU scenarios exhibited a dramatic recovery in percent total lipids during the observational period (Fig. 2e, Table 2d).

### Chlorophyll fluorescence

**Figure 4:**
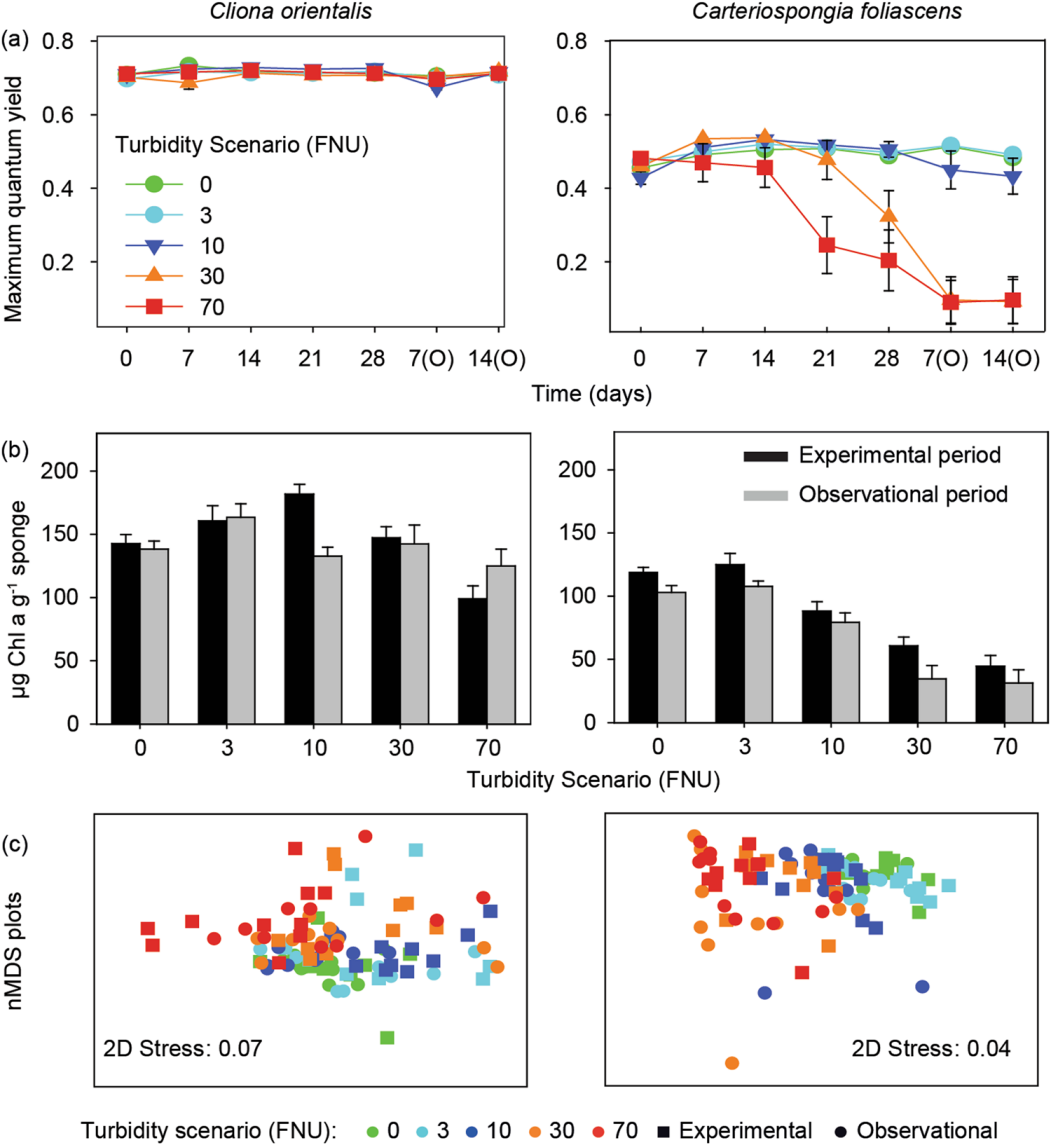
Photosymbiont response to the 5 different scenarios. (**a**) Mean values (±SE) of maximum quantum yield, (**b**) Mean values ( ± SE) of Chl *a*, and (**c**) Non-metric Multi-Dimensional Scaling (nMDS) of all photopigments retrieved by spectrophotometry, for the two phototrophic species and for all scenarios in the experimental and observational phases.

Maximum quantum yields were highly stable among scenarios in the phototrophic sponge *C*. *orientalis* throughout the experimental and observational phases (Fig. 4a, Table 4a). In *C*. *foliascens*, maximum quantum yields were significantly lower in bleached (discoloured) individuals from day 21 and 28 under the 70 and 30 FNU scenarios, respectively (Fig. 4a, Table 4a). High rates of bleaching, necrosis and subsequent mortality precluded recovery in this species.

**Table 4:** Analyses of variance on the effects of turbidity scenarios on photosymbionts.

Parameter	Phase	Source	*df*	*Cliona orientalis*		*Carteriospongia foliascens*	
				*F*	*P*	*F*	*P*
(**a**) maximum quantum yield	Expt.	Scenario	4	2.920	0.031	7.338	<0.001
		Error	45				
		SNK		10 > 30		0, 3, 10 > 70	
	Obs.	Scenario	4	0.914	0.464	20.824	<0.001
		Error	45				
		SNK				0, 3, 10 > 30, 70	
(**b**) Chl *a*	Expt.	Scenario	4	10.520	<0.001	23.286	<0.001
		Error	45				
		SNK		3, 10 > 30 > 70		0, 3 > 10 > 30, 70	
	Obs.	Scenario	4	1.726	0.161	20.077	<0.001
		Error	45				
		SNK				0, 3 > 10 > 30, 70	
(**c**) PERMANOVA of all pigment data	Expt. + Obs.	Scenario	4	5.4241	0.0001	24.9	0.0001
		Time (Scenario)	5	3.6263	0.0009	2.3848	0.0071
		Error	90				
		Pair-wise Tests		0 ≠ 3, 10, 30 ≠ 70		0, 3 ≠ 10 ≠ 30, 70	

One-way ANOVA examining the effects of turbidity scenarios on (**a**) maximum quantum yield and (**b**) Chl *a* concentrations, at the end of the experimental and observational periods, with Student Newman-Keuls (SNK) tests performed for significant pairwise multiple comparisons, and (**c**) Two-way PERMANOVA of all pigment data (Chl *a*, *b*, *c*, *d*, total Chlorophyll and Carotenoids) with scenario and time as factors, for the two phototrophic species (scenarios: 0, 3, 10, 30 and 70 FNU).

### Pigment analysis

Chl *a* concentration was highly correlated to total chlorophyll in both *C*. *orientalis* and *C*. *foliascens* (R^2^ = 0.997 and 0.998 and *P* < 0.001, respectively). In addition, Chl *a* was highly correlated to Chl *c* in *C*. *orientalis* and negatively correlated to Chl *d* in *C*. *foliascens* (R^2^ = 0.993, −0.392 and *P* < 0.001, respectively). Overall, concentrations of Chl *a* were stable throughout the experiment in the controls and lower turbidity scenarios (≤3 FNU) (Fig. 4b). However, significantly lower concentrations of Chl *a* were observed under higher turbidity scenarios (≥30 FNU) at the end of the experimental period for both species (Fig. 4b, Table 4b). Accordingly, Chl *c* in *C*. *orientalis* also decreased significantly in the 70 FNU scenario (ANOVA: *P* < 0.001) at the end of the experimental period (Supplementary Fig. S2). At the end of the 14 d observational period, *C*. *orientalis* did not show any significant differences between scenarios, consistent with a recovery of its original health status, colour and quantum yields after temporary bleaching (Fig. 4b, Table 4b, Fig. S2). *C*. *foliascens* did not recover during the observational period, and significantly lower values of Chl *a* were retrieved from scenarios ≥ 10 FNU (Fig. 4b, Table 4b). However, the negative correlation between Chl *a* and Chl *d* in *C*. *foliascens*, and a significant increase in Chl *d* in samples exposed to ≥ 30 FNU scenarios (ANOVA: *P* < 0.001), suggests an increase in some Chl *d*-containing Cyanobacteria under the higher turbidity scenarios (Fig. S2).

Non-metric Multi-Dimensional Scaling (nMDS) analysis of normalized data for all pigments retrieved by spectrophotometry (Chl *a*, Chl *b*, Chl *c*, Chl *d*, Total Chlorophyll and Carotenoids) showed some grouping of the samples according to scenarios for both species (Fig. 4c). This was most evident for *C*. *foliascens* exposed to ≥ 10 FNU (Fig. 4c), which is consistent with patterns observed for Chl *a*. PERMANOVA analysis confirmed significant differences between scenarios in both species, with subsequent pair-wise testing showing main differences between low and high turbidity scenarios (Table 4c).

## Discussion

Turbidity associated with the release of sediments into the water column by dredging or natural resuspension can affect the sponge holobiont in many ways, primarily involving suspended sediment, light attenuation and sediment deposition. These stressors can act either alone or, more frequently, in combination and can change rapidly according to sea-state, cloud cover, and diel and tidal cycles^4, 32^. This ‘protean’ characteristic of turbidity makes it very difficult to predict impacts for management purposes. The results from this study show a range of responses from three sponge species to the combined effects of elevated SSC, light attenuation and sedimentation, with sponge health being negatively affected by moderate to high turbidity scenarios (≥10 mg L^−1^, ≤0.8 mol photons m^−2^ d^−1^). Responses included the production of mucus-like substances, bleaching, oscula (excurrent openings) closure, lipid depletion and tissue regression. Reduced chlorophyll content and low maximum quantum yield under the highest turbidity scenarios (≥33 mg L^−1^, ≤0.5 mol photons m^−2^ d^−1^) also indicated negative effects on the photosymbionts within the phototrophic sponges. In fact, for the phototrophic species, when light reduction and elevated suspended sediments were combined, it resulted in greater sub-lethal and lethal effects than seen previously when the stressors were applied in isolation^23, 34–36^. In addition, mortality occurred in *C*. *foliascens* and *C*. *orientalis* under the highest turbidity scenario (90% and 20%, respectively), although most *C*. *orientalis* and all individuals of *I*. *basta* survived and recovered once returned to clear water conditions.

The response of the sponges to the combined stressors and even to isolated stressors in the studies described in^34–36^ was varied and particular to the sponge species and nutritional mode being examined. Elevated turbidity caused some sponges, especially *C*. *foliascens*, to produce mucus-like sheets which trapped sediments. The sheets eventually sloughed off the surface into the water column removing sediments from the sponge. The term mucus has been used here in a broad sense^37^, as the composition of the sponge mucus layer has not been characterised. Production of mucus-like substances in sponges has been anecdotally noted before in *Hemectyon felix* ^25^, *Crambe crambe* ^38^, *Mycale acerata* ^39^, *Rhopaloeides odorabile* ^40^, *Carteriospongia foliascens*, *Coscinoderma matthewsi*, *Cymbastela coralliophila* and *Stylissa flabelliformis* ^36^. The formation of mucus sheets has been previously reported in hard corals, particularly from the genus *Porites* ^41, 42^. A number of studies have inferred this plays a role in self-cleaning in *Porites* spp. to combat sedimentation, although others have suggested this is a secondary phenomenon^42, 43^. Certainly for species such as *C*. *foliascens* and *I*. *basta* the episodic shedding of sediment-impregnated mucus-sheet was an effective mechanism to remove surface accumulated sediments (Fig. 3b). As also noted for corals, the production of the layer is expected to come at an energetic cost^44^, which would make it a difficult process to sustain during chronically elevated suspended sediments loads^11, 35^ and/or repeated smothering^36^.

To our knowledge, this is the first study to quantify the production of the mucus-like substance over time in sponges, and specifically, in response to sediment exposure. *C*. *foliascens* and *I*. *basta* both produced mucus layers under all sediment treatments (≥3 mg L^−1^), although *C*. *foliascens* showed the highest percentage of mucus cover (i.e. up to 80%) from the start of the experiment. *C*. *foliascens* also experienced a depletion in energy reserves and high mortality towards the end of the exposure, which may be due to an inability to maintain phototrophic feeding under low light conditions (as suggested by decreased Chl *a* and reduced maximum quantum yields under the high turbidity scenarios). These results are consistent with previous research reporting that *C*. *foliascens* is unable to shift between nutritional modes (i.e. increasing heterotrophic feeding), and its intimate and potentially obligate symbioses with *Cyanobacteria* is adversely impacted by high SSCs and light attenuation^34, 35^. Therefore, formation and sloughing of mucus sheets in *C*. *foliascens* may only be effective under low SSCs with limited light attenuation or as a short-term response (<7 d) under higher turbidity scenarios before irreversible effects occur.

*C*. *orientalis* showed a significant reduction in the number of open oscula under high SSCs, with total oscula closure in the highest turbidity scenario. Oscula closure in this species is strongly correlated with reduced pumping^28^, which is a common response of sponges to elevated SSCs^25, 26, 28, 35, 39, 45, 46^. This response can be effective in the short term as it prevents clogging of the aquiferous system. However, it can also adversely affect filter-feeding, reducing food-retention efficiency and potentially leading to starvation in obligate heterotrophs^35, 39, 40^. Increased oscular closure together with potential canal blockage may explain the mortality of 20% of *C*. *orientalis* individuals in this study and is likely to be a response to the suspended sediment concentration.

A common feature in the higher turbidity scenarios in the phototrophic species was sponge bleaching or tissue discolouration concomitant with reductions in chlorophyll concentration. This was often a sublethal response, and in many cases sponges regained their pigmentation in the post exposure observation period. However, in the high turbidity scenarios, bleaching and partial and whole sponge mortality were intimately linked. Bleaching in these species has been reported previously solely in response to light deprivation^34^, and the response is most likely a function of the low light levels associated with the turbidity scenario, as opposed to the high SSCs.

In addition to the production of mucus layers, *I*. *basta* also underwent tissue regression in response to high SSCs, which reduces the number of choanocyte chambers and results in more densely packed cells^47, 48^. This mechanism for coping with sediment stress reduces the risk of the aquiferous system becoming clogged, but also causes the sponge to enter a stress-induced ‘dormant’ state^47^. This dormant state may not be energetically sustainable in the long term as suggested by the reduction in total lipids in *I*. *basta* under high turbidity scenarios. Hence, although all 3 species possess mechanisms to cope with different sediment-related stressors, and despite the ability of both *C*. *orientalis* and *I*. *basta* to survive and recover from sediment stress in this 28 d experiment, we hypothesize that all 3 species would undergo higher mortalities if the stressors persist in time.

The development and use of sub-lethal stress indicators would alert dredging proponents of water quality conditions before they could detrimentally impact sponge populations. Discolouration and necrosis of sponge tissue have been previously described as effective bioindicators for dredging related stress^35^. However, discolouration could also be related to natural causes or diel patterns in some species, such as the day-night migration of *Symbiodinium* sp. in *C*. *orientalis* ^49^ which would require the use of this bioindicator with caution and at defined times. On the other hand, lesion formation (i.e. partial mortality) may be irreversible in some sponge species and lead to rapid mortality as observed here and in previous studies for *C*. *foliascens* ^34, 35^. Although it is challenging to identify universal bioindicators that could be applied to all sponges, our results suggest that mucus sheet production, oscula closure and tissue regression could be effective bioindicators of turbidity-related stress in some sponge species and could be incorporated into future sponge monitoring programs. Mucus excretion has previously been proposed as a useful indicator for sediment stress in sponges^10, 11^ and corals^50, 51^ and tissue regression has previously been described as a symptom of stress^47^.

The response of some of the species to the combined effects of elevated suspended sediment and reduced light availability was more immediate and severe than when applied alone in previous studies conducted under the same or similar conditions and species^34, 35^. Specifically, decreased light levels coupled with high SSCs affected both phototrophic and heterotrophic feeding strategies simultaneously and likely contributed to the earlier and higher mortalities observed in *C*. *foliascens* and *C*. *orientalis* in comparison to previous studies assessing these factors independently^34, 35^. For example, in this study, exposure of *C*. *foliascens* to high SSCs (33 mg L^−1^) in combination with low light (0.5 mol photons m^−2^ d^−1^) resulted in tissue bleaching and 55% mortality within 28 d. In contrast, exposure to 0.8 mol photons m^−2^ d^−1^ (with a SSC of 0 mg L^−1^) did not cause any mortality^34^, and exposure to 23 mg L^−1^ (with a DLI of 5 mol photons m^−2^ d^−1^) resulted in lower mortality^35^ (Supplementary Table S1). Similarly, exposure to 76 mg L^−1^ in combination with a DLI of 0.15 mol photons m^−2^ d^−1^ resulted in tissue bleaching and 85% mortality within 7 d, whilst exposure to 73 mg L^−1^ alone resulted in mortality only after 14 d, and 0 mol photons m^−2^ d^−1^ alone resulted in mortality only during the recovery period (>28 d) (Supplementary Table S1). There was some sediment deposition on the sponges in this study which is another potential known stressor; however, the accumulation rate of sediment was low, reaching up to 5.2 mg cm^−2^ d^−1^. In previous studies, exposure to repeated sediment deposition events of up to 44 mg cm^−2^ for over a similar period (28 d) did not result in any bleaching or mortality in a range of species, including *C*. *foliascens* ^36^. For *C*. *orientalis*, it occasionally resulted in small accumulations of sediment in local surface depressions which led to small bleached patches and eventually to lesions. Although deposition rates in the present study were lower, similar bleached patches were observed where sediments accumulated.

It is possible to place the results of this study and previous studies^34, 35^ into context, using recent analyses of water quality monitoring data from several large scale dredging projects in tropical waters^5, 6, 33^. The impacts of dredging on light attenuation and SSCs followed a power-law decay relationship, with sites near the excavation activities experiencing greater changes to water quality than more distant ones^5^. One of the dredging campaigns was conducted in a clear-water environment (Barrow Island, Western Australia^4^), with similar water quality to the collection location of sponges in this study. The average nephelometrically-derived SSCs and DLIs were calculated for five sites located <1 km from the dredging^5^. Over 30 d running mean periods determined for the duration of the 1.5 year program, the *P*_5_ of mean DLIs was 0.4 mol photons m^−2^ d^−1^, as opposed to 1.9 mol photons m^−2^ d^−1^ during the baseline phase. Based on the laboratory studies examining the effects of light reduction alone over a similar period, a DLI of 0.8 mol photons m^−2^ d^−1^ resulted in bleaching in *C*. *orientalis* and *C*. *foliascens* ^34^. During the dredging, the *P*_95_ of SSCs over a 30 d running mean period was 23 mg L^−1^, as opposed to 3.2 mg L^−1^ during the baseline phase. The laboratory based studies, examining the effects of elevated SSCs alone over a 30 d period, showed a range of effects at ≥23 mg L^−1^ and few negative effects at SSC of ≤10 mg L^−1^ ^35^. Both of these laboratory-based studies suggest that even when applied in isolation, exposure to environmentally realistic conditions (of light reduction and elevated SSCs during dredging) could have negative effects on sponge communities. The results from this study, suggests that combinations of high SSCs and low light availability can accelerate and increase mortality increasing the probability of biological effects.

Finally, although sponges have mechanisms or adaptations to cope with dredging-related pressures in the short term, these tolerance mechanisms come at a cost, as evidenced by reduced lipids and deterioration of sponge health in all species towards the end of the experiment, suggesting that longer term exposure to similar conditions is likely to result in higher mortality. The LC_50_ and LC_10_ values derived for *C*. *foliascens* in this study and a previous study^35^ could be used by managers and dredging proponents when implementing zones of impact based on dredge plume models. Priorities for future research include (i) assessing longer term impacts, (ii) assessing the effect of different frequency events and (iii) assessing the effects on potentially sensitive early life history stages.

## Methods

### Sample collection

Three sponge species, representing three general morphologies (encrusting, cup and fan) and nutritional modes (i.e. phototrophic and heterotrophic), were collected from 3−15 m depth from the Palm Islands, central Great Barrier Reef (GBR) in March 2016 (Table 5). *Cliona orientalis* (Thiele, 1900), *Carteriospongia foliascens* (Pallas, 1766) and *Ianthella basta* (Pallas, 1766) are common throughout the Indo-Pacific, including the east and west coasts of tropical Australia^52^. At least one quarter of each sponge individual was left in the field in order to facilitate recovery^53^. *C*. *foliascens* and *I*. *basta* were cut into large explants (~5 × 5 cm) and cores (radius: 2.45 cm, height: 2 cm) of the encrusting sponge *C*. *orientalis* were drilled from dead colonies of *Porites* spp. Sponges were transported to the Australian Institute of Marine Science (AIMS, Townsville) and acclimated for two weeks under natural light conditions in a 5000 L tank with flow-through seawater at 28 °C and 36‰ salinity. Experiments were conducted with calcareous sediment collected from the lagoon of Davies Reef, a mid-shelf reef centrally located in the GBR (S 18° 49.354′ E 147°38.253′) and processed to a predominately silt-size typical for dredge plumes^54^ (mean particle size of 29 µm, range: 3-64 µm) as described in^35^.

**Table 5:** List of sponge species, morphologies, nutrition mode and sampling locations.

Species Name (Author)	Functional Morphology	Primary Nutritional Mode	Sampling location
*Cliona orientalis* (Thiele, 1900)	Encrusting (bioeroding)	phototrophic^55^	Pelorus Is. (Palm Is.) S 18°32.903′ E 146° 29.172′
*Carteriospongia foliascens* (Pallas, 1766)	Cup (wide cup)	phototrophic^56^	Fantome Is. (Palm Is.) S 18°41.028′ E 146° 30.706′
*Ianthella basta* (Pallas, 1766)	Erect (laminar)	heterotrophic^57^	Fantome Is. (Palm Is.) S 18° 42.291′146° 30.591′

### Experimental set up

Experiments were conducted in 10 × 1200 L fibreglass tanks with 5 µm filtered seawater in an environmentally controlled room within the National Sea Simulator (SeaSim) at AIMS (see Supplementary Fig. S3). Sponges were placed on a false bottom floor made from fibre reinforced plastic grating (80% open) at a depth of 50 cm below the surface. Each tank had a Perspex window for observations during the experiments.

Sponges were exposed to 5 different situations representing different intensities of increased suspended sediment concentrations (SSCs), and associated light attenuation and sedimentation rate (see Table 1). Hereafter, the different treatments are referred to as ‘scenarios’ and based on the nominal turbidity (0, 3, 10, 30 and 70 NTU) (Table 1). Two tank replicates were used for each scenario, with 10 replicate sponges per species in each tank. As replicate tanks within each treatment behaved similarly throughout the experiment for all physical parameters (i.e. turbidity, light and sediment deposition, T-tests: *P* > 0.05), and sponges were unlikely to influence each other within the large 1200 L tanks (i.e. sponges were >20 cm apart), sponges rather than tanks were used as replicates for statistical analyses. Sponges were acclimated to experimental control conditions in the tanks for one week prior to commencing the experiment. The experiment ran for a 28 d ‘experimental period’ followed by a 14 d ‘observational’ period where all sponges were returned to sediment-free seawater (Table 1).

The SSCs and associated light combinations were based on recently reported data from several large scale capital dredging programs^5, 6^ (Table 1). Because the experiments were conducted in shallow containers (with only 50 cm water depth), the light attenuation associated with a given SSC would be much less than would occur *in situ* on a typical reef and it was therefore necessary to adjust the light accordingly. We nominally chose a water depth similar to the sponge collection depth (7 m) and used the relationships between water depth, SSC and PAR described in^6^ (which was based on empirical data collected by multiple light profiles through a dredging plume) to simulate the maximum down welling irradiance for a clear (cloud-free) sky at solar noon (with an initial underwater, 0 m depth PAR value of ~1530 μmol photons m^−2^ s^−1^)^6^. During cloudy days, at lower azimuth angles and at different sea states, underwater light quality and quantity would differ substantially.

Lighting was provided by 2 custom-made sets of Light Emitting Diodes (LED) positioned above each tank. Light within each tank was measured using a photosynthetically active radiation (PAR) Quantum Sensor (Skye, UK) positioned on the fiber reinforced plastic grating next to the sponges. Both the lights and the light sensor were connected to a programmable logic controller (PLC) system which controlled the light based on the SSC (see above). In addition, light intensity in each tank simulated daylight variation, with a 6 h ramping up from 05:30 h (sunrise) to full light from 11:30−12:30, followed by a 6 h ramping down to sunset at 18:30 h. Light levels were expressed as the daily light integral (DLI) in units of mol photons m^−2^ d^−1^, which is the sum of the per second quantum flux measurements over the course of 24 h.

Water flow into the tanks was standardized to 1000 mL min^−1^ to ensure ~1.5 complete turnover per day. Water temperature was 28 ± 1 °C in all tanks over the duration of the experiment. SSCs were mixed within the experimental tanks using a recirculating Iwaki MX pump (Iwaki Co., Ltd., Japan) at 45 Hz and an underwater Hydrowizard pump (Panta Rhei, Germany) which was set to oscillate from 24−25% power in ‘wave’ mode (0.6 s pulse: 0.6 s no pulse). The combined pumps created a turbulent, flow typical on shallow coral reefs^58^, generating in-tank flow rates of ~4 cm s^−1^, as measured using a mini acoustic doppler velocimeter (ADCP, SonTek 16-MHz MicroADV Sontek, US). To bring the SSC to the desired levels and to replace sediment lost from the tanks (by the water exchange) short pulses of a high SSC (~6 g L^−1^) sediment slurry from a 500 L stock tank was episodically injected into the tanks through a solenoid valve. SSCs were continually measured in each tank using turbidity as a proxy, using Turbimax CUS31 nephelometers (Endress and Hauser, Germany) connected to the PLC system. Nephelometers were calibrated to formazin nephelometric units (FNU), and a near 1:1 correlation was established between FNU and mg L^−1^ of sediment (see equation below). SSCs in the tanks were kept constant by a continuous feedback mechanism from the nephelometer probes through the PLC which also controlled the opening and closing of the solenoids as required. Associated with the SSC, and corresponding light level, there was also some sedimentation that occurred on the sponges in the tanks (Table 1).

### Physical parameters

Water samples (3 × 200 mL) were collected from each tank each week and the SSCs were determined gravimetrically by filtering the samples through pre-weighed 0.4 µm polycarbonate filters, drying the samples (60 °C for 24 h) and re-weighing the filters.

Throughout the experiment, FNU values related to SSCs according to the following equation:

$${\rm{SSC}}({\rm{mg}}\,{{\rm{L}}}^{-1})=1.089\times {\rm{FNU}}({{\rm{F}}}_{1,9}=578.5,P < 0.01,{{\rm{R}}}^{2}=0.985)$$

Sediment deposition was measured in real-time using a deposition sensor^59^ located in the middle of the grate floor and two SedPods^60^ (Surface Area = 25.2 cm^−2^) randomly placed in each tank. SedPod sediment accumulation rates were measured weekly by collecting and filtering sediments that settled on the surface for 24 h.

Differences in turbidity and DLIs were analysed between replicate tanks with T-tests and with a one-way repeated measures analysis of variance (ANOVA) at the end of the experimental and observational phase with treatment as fixed factor. Differences in SSCs and SR between treatments were furthered assessed throughout the experiment with a two-way repeated measures ANOVA using treatment (i.e. scenario) and day as fixed factors.

Unless otherwise stated, statistical analyses and graphs were performed using the software R v. 3.1.0^61^ and SigmaPlot v.11.0 (Systat Software Inc.).

### Studied parameters

After the 28 d experimental period, 10 individuals from each species were sampled from each scenario (and removed from the experiment), and a further 10 individuals were sampled after the 14 d observational period, for all ‘destructive’ analyses (i.e. lipids and pigments). To obtain baseline data on sponge health, 5 extra individuals were processed for each species after aquarium acclimation (t = 0 controls).

In order to enable weekly ‘non-destructive’ measurements (i.e. pictures, visual assessments and fluorescence measurements), sediment dosing was interrupted and the power in the Iwaki and Hydrowizard pumps decreased to 20 Hz and 1% respectively, causing a subsequent drop in turbidity levels in the tanks for a period of < 20 h from 16.00 h on Tuesday until 12.00 h on Wednesday, each week. Light values, however, were maintained at the corresponding levels irrespectively of SSCs to minimize effects on the experimental scenarios.

### Sponge health, growth and stress responses to sediments

A 2-dimensional approach for growth (i.e. surface area), partial mortality (necrosis), tissue regression and loss of photosynthetic symbionts (discolouration or bleaching) were recorded weekly using a digital camera and analysed using image analysis software (ImageJ^62^). Growth and bioerosion in *C*. *orientalis* cores was determined using buoyant weights (±0.001 g) before and after the experimental and observational periods, and normalized to surface area to obtain changes in biomass per unit area, which are correlated to bioerosion rates in this species^63^. Relative growth rates in *C*. *foliascens* and *I*. *basta* were calculated as the log_10_ of their final surface area (SA) measure divided by their initial SA.

In addition, sponges were visually assessed at midday once a week to check for stress responses, including percent mucus cover, sediment cover, bleaching and necrosis. Sponges presenting >50% necrosed tissue were considered effectively dead and were sampled for ‘destructive’ analyses. The percentage of tissue regression was also assessed in *I*. *basta*, which presents a dynamic pattern of regression/recovery^47^. The contractile behaviour of the oscula in *C*. *orientalis* was used as an additional stress response. This involved comparing the numbering of open oscula to the total number of oscula per individual throughout the experiment.

All treatment effects were tested with one-way ANOVAs; statistical analyses found that there was no significant difference between tanks within scenarios, thus tank effect was not considered further. In any instances where homogeneity of variances and normality were not met, a Kruskal-Wallis one-way ANOVA on ranks was performed. Mortality data was fitted to nonlinear regression curves using the program Prism v7.01 (GraphPad Software Inc, US). Regression curves were used to calculate the lethal concentrations (LC) of SSC and DLI at which 50% (LC_50_) and 10% (LC_10_) of the population died. The models were constrained between 0 and 100 with F values set at 50 and 10, for LC_50_ and LC_10_, respectively. The curve was tested for normality of the residuals and a replicate test was applied to assess goodness of fit. Asymmetrical confidence intervals were calculated for the LC values.

### Lipid analysis

The concentration of total lipids in sponge tissue was measured over time as a proxy for health (i.e. energetic stress). Samples were analysed at the start of the experiment and at the end of the experimental and observational periods in the 0, 30 and 70 NTU scenarios. Lipids were extracted from ~100 mg of freeze-dried ground sample as described elsewhere^35, 64, 65^. Total lipid content was reported as percentage biomass based on a dry weight conversion factor. T-tests were used to assess changes in lipid content throughout the experiment in control samples, whereas differences between scenarios at the end of each sampling point were assessed for each species separately using a one-way ANOVA.

### Chlorophyll fluorescence

Photosynthetic capacity (maximum quantum yield) of the phototrophic symbionts in *C*. *orientalis* and *C*. *foliascens* was measured with a Diving-PAM (pulse amplitude modulation) chlorophyll fluorometer (Heinz Walz GmbH, Effeltrich, Germany) as described in^34^. Briefly, maximum quantum yield (*F*_v_/*F*_m_)^66^ measurements were obtained from dark-adapted sponges (measured before sunrise) at weekly intervals throughout the experimental and observational periods. Bleached individuals of *C*. *foliascens* that died during the experiment were assumed to have the lowest detected quantum yield value (zero) in subsequent weeks after mortality throughout the time series. Changes in maximum quantum yield at the end of the experimental and observational periods were assessed for each species separately using a one-way ANOVA with scenario as the fixed factor.

### Pigment analysis

Pigment analyses were performed on tissue from the phototrophic sponges *C*. *orientalis* and *C*. *foliascens* at the end of the experimental and observational periods. Pigments from samples incorporating pinacoderm and mesohyl regions were extracted and analysed as described in^23^ and standardized to sponge wet weight. The concentration of Chlorophyll *a* (hereafter Chl *a*) was used as a proxy for changes in photosymbiont health/activity (i.e. bleaching)^15^. Changes in Chl *a* concentration at the end of the experimental and observational periods were assessed for each species separately using a one-way ANOVA with scenario as the fixed factor. Pearson correlations were performed between all studied pigments. All pigments measured by spectrophotometry (i.e. chlorophylls *a*, *b*, *c*, *d*, Total chlorophylls and carotenoids) were used to build resemblance matrices based on normalized data for each species separately. Non-metric Multi-Dimensional Scaling (nMDS) plots were created using Euclidean distances. Two factors were determined (i.e. scenario and sampling time, nested to scenario) and examined by PERMANOVA (Permutational multivariate ANOVA based on distances). All multivariate analyses were performed using Primer 6 (Primer-E Ltd, UK).

## Electronic supplementary material

Supplementary Information
