# Supplementary material for: Effects of combined dredging-related stressors on sponges: a laboratory approach using realistic scenarios
Source: Sci Rep. 2017 Jul 12;7:5155. doi: 10.1038/s41598-017-05251-x (PMC5507900; doi:10.1038/s41598-017-05251-x)
Supplement: Supplementary file 1 — Supplementary Information [file 41598_2017_5251_MOESM1_ESM.pdf]

Supporting Online Material

Effects of combined dredging-related stressors on sponges: a laboratory approach using realistic scenarios

Mari-Carmen Pineda[1,2^*], Brian Strehlow[3^], Jasmine Kamp[4], Alan Duckworth[1,2], Ross Jones[1,2] and Nicole S. Webster[1,2]

[1] *Australian Institute of Marine Science (AIMS), Townsville, QLD and Perth, WA, Australia*

[2] *Western Australian Marine Science Institution, Perth, WA, Australia*

[3] *Centre for Microscopy Characterisation and Analysis, School of Plant Biology and Oceans Institute, University of Western Australia, Crawley, WA, Australia*

[4] James Cook University, Townsville, QLD, Australia

^represents joint 1st authors

*Corresponding author:

Mari-Carmen Pineda

Australian Institute of Marine Science, PMB3, Townsville, QLD, 4810, Australia

E-mail: mcarmen.pineda@gmail.com.

Tel.: +61 7 4753 4522, fax: +61 7 4772 5852

Figure S1. Percentage of mortality for each scenario across the experiment in *C. orientalis* and *C. foliascens*.

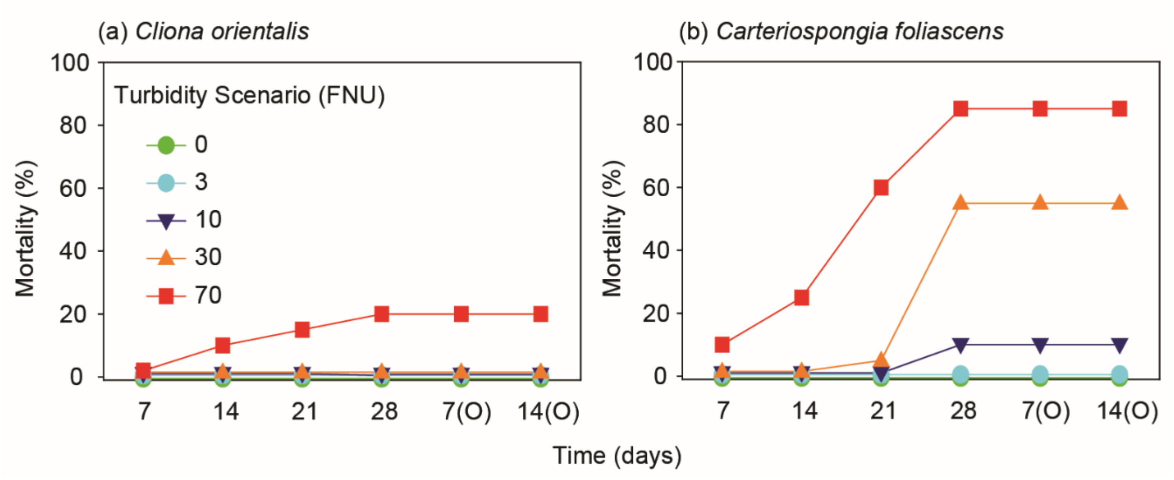

Figure S2. Mean values (±SE) of (a) Chl c and (b) Chl d in *C. orientalis* and *C. foliascens*, respectively, at 5 turbidity scenarios after the experimental and observational periods.

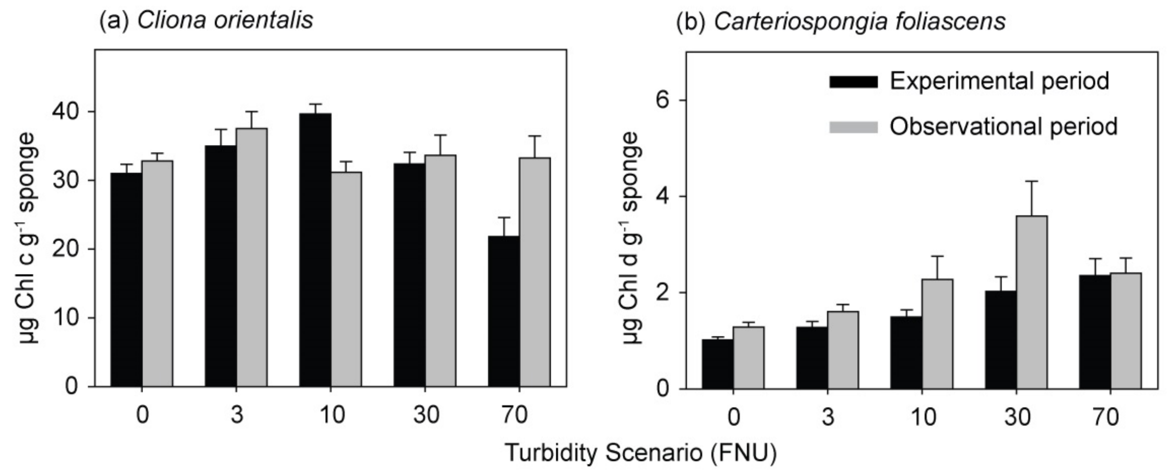

Figure S3. Experimental set up. Experimental set up in an environmentally controlled room within the National Sea Simulator (SeaSim) at the Australian Institute of Marine Science (AIMS, Townsville, Australia).

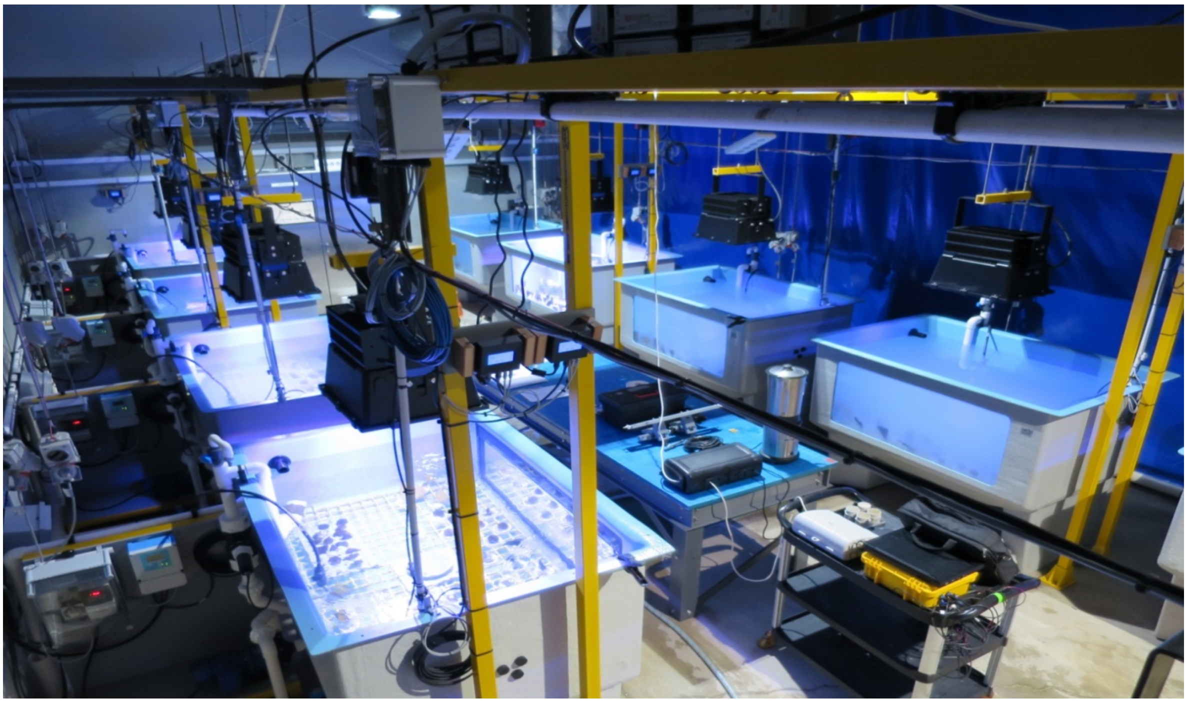

Table S1. Response of phototrophic sponges to stressors in isolation and combined. Response of the boring sponge *C. orientalis* and the foliose sponge *C. foliascens* to light reduction (as daily light integral [DLI], mol photons m^{-2} d^{-1}) and elevated SSCs (nephelometrically-derived SSC, mg L^{-1}) alone or in combination over a 28 d experimental exposure period (exp.) and subsequent 14 d observational period (obs.).

Experiment (Source)	SSC (mg L^{-1})	Light (DLI) (mol photons m^{-2} d^{-1})	*Cliona orientalis*	*Carteriospongia foliascens*
SSC constant light variable ([34])	0	0	Bleaching (>3 d) but no mortality	Bleaching (>7 d). No mortality in exp. 100% mortality during obs.
	0	0.8	Minor bleaching at end of the 28 d exp. but no mortality	Minor bleaching towards the end of exp. but no mortality
Light constant SSC variable ([35])	10	5	~5% bleaching (28 d) but no mortality	5–20% bleaching (>21 d) and 20% mortality (from 28 d)
	23	5	5–10% bleaching in exp. but no mortality	5–20% bleaching (>21 d) and 20% mortality (>28 d)
	73	5	40% bleaching (>7 d) but no mortality	40–80% bleaching (>14 d) and 90% mortality (>14 d)
Light variable SSC variable (this study)	11	0.87	~10% bleaching (>21 d) but no mortality	20% bleaching (>14 d) and 10% mortality (>21 d)
	33	0.5	20% bleaching (>14 d) but no mortality	90% bleaching (>14 d) and 55 % mortality (>14 d)
	76	0.15	60% bleaching (>14 d), 20% mortality (> 14 d)	90% bleaching (>7 d) and 85% mortality (>7 d)
